# Suicide in South India: A community-based study in Kerala

**DOI:** 10.4103/0019-5545.58290

**Published:** 2009

**Authors:** C. R. Soman, S. Safraj, V. Raman Kutty, K. Vijayakumar, K. Ajayan

**Affiliations:** Health Action by People, TC 7/1724, Temple Road, Kochulloor, Trivandrum, Kerala - 695 011, India

**Keywords:** Kerala, South India, suicide

## Abstract

**Background:**

Studies from Tamil Nadu, South India, have reported the world's highest suicide rates. As per official reports, Kerala, another South Indian state has the highest suicide rate among the major states in India.

**Objective:**

The purpose of this analysis is to estimate the rates and age-specific incidence of suicide in a rural community in Kerala, under continuous observation for the last five years.

**Settings and Design:**

The study setting comprised of seven contiguous panchayats constituting a development block in Kerala. A prospective cohort study design was used.

**Materials and Methods:**

Through regular home visits, every death that occurred in the community was captured by local resident health workers and the cause of death assigned.

**Statistical Analysis:**

Suicide rates by age and sex and relative share of suicide deaths to all-cause deaths in men and women were calculated.

**Results:**

During the five-year period from 2002 to 2007, 284 cases of suicide were reported. The suicide rates were 44.7/100,000 for males and 26.8/100,000 for females. Male to female suicide ratio was 1.7. Among females aged between 15 and 24, suicides constituted more than 50% of all deaths. Male to female ratio of suicide varied from 0.4 in children aged 14 years or less to 4.5 in the 45-54 year age group.

**Conclusion:**

Our analysis shows that the level of under-reporting of suicides in rural Kerala is much less than that reported in Tamil Nadu.

## INTRODUCTION

In the year 2000, approximately one million people died the world over, as a result of suicide.[1] In India, according to government estimates, 113,914 people committed suicide in the year 2005.[2] The national suicide rate for the year 2005 was 10.3/100,000 and it varied from 0.6/100,000 to 52.1/100,000 in different parts of India.[2] These rates, calculated from police records, are likely to be underestimates. Societal stigma, legal consequences of the reporting of suicide, and estimation of suicide data using police records are known to be probable causes for the under-reporting of suicides in India and elsewhere.[3]

According to the National Crime Record Bureau, the official agency responsible for suicide data collection in India, Kerala, a South Indian state, has the highest suicide rate among Indian states. In 2005, Kerala had an estimated suicide rate of 27.7/100,000. In that year, suicide rates in the other South Indian states were: Karnataka 20.7/100,000, Tamil Nadu 18.6/100,000, and Andhra Pradesh 16.8/100,000. The union territory of Pondicherry had the highest suicide rate in India at 52.1/100,000. In comparison with the south, suicide rates reported from other parts of India were low: Delhi, the national capital had a suicide rate of 7.9/100,000, while the reported rates from Gujarat, Nagaland, and Bihar were 8.8, 1.3, and 0.6, respectively, per 100,000 persons.[2]

The most notable community-based studies on suicide in India have emerged from the south Indian state of Tamil Nadu. In a series of articles based on a prospective community study run by the Christian Medical College, Vellore, in Kaniyambadi block, the authors reported very high rates of suicide in the study area, 137/100,000.[4–6] Gajalakshmi and Peto after analyzing 38,386 deaths using verbal autopsy techniques in Villupuram district, reported that intentional self-harm was responsible for 47.8% of the deaths, due to external causes. The estimated combined suicide rate was 62/100,000.[7] Both studies also reported a very high rate of suicide among young women aged between 15 and 24 years. These studies cast a shadow on the reliability of the official suicide statistics. The rates reported in these studies are the highest in the world and South India has subsequently been described in the media as the ‘suicide capital of the world’.[8,9]

The purpose of this analysis was to estimate the rates and age-specific incidence of suicide among males and females in a rural community in Kerala, under continuous observation for the last five years.

## MATERIALS AND METHODS

The data reported here is from the PROLIFE study, a prospective cohort study involving a long-time follow-up of the residents of seven villages in Kerala.[10] The immediate objective of the PROLIFE study was to set up a community-based registry for lifestyle diseases and to reliably assess the cause of deaths in the cohort. The present data on suicide was extracted from the data on deaths over a five-year period. Health workers numbering 105, employed by the local government, were used for data collection. The health workers were given a thorough training on the data collection techniques by a team of doctors and public health experts. The training involved 12 sessions of 90 minutes duration followed by supervised field level data collection. A simplified, structured questionnaire, based on a World Health Organization (WHO) verbal autopsy instrument was provided to each worker to assist in the assignment of the cause of death.[11] The instrument was developed primarily with a focus of identifying cardiovascular disease (CVD)-related deaths in the community. Our questionnaire contained additional questions to capture non-cardiac deaths. The health workers were residents of the community and got to know of any death in the community within a short span of time. The members of the community and the local government officials were advised to report any death to the health workers with minimal delay. On almost all occasions, a reported death was investigated within two weeks of its occurrence. All causes of death reported by the field workers were reviewed by a physician in the study center. The physician assigned a cause of death to each event on the basis of the tenth revision of the International Classification of Diseases (ICD).[12]

The physician conducted a validation study during the first year of data collection (2002-2003) and analyzed 647 deaths reported by the health workers. He visited the households and made a detailed inquiry into the cause of death reported by the health worker, using the previously referred questionnaire.[11] The physician also sought and used available documents pertaining to hospitalization or treatment. He then assigned a cause to every death. The principal investigators regularly met with the physician and reviewed each reported cause of death. The final cause of death was assigned during this meeting and was used as the standard against which the reports of the health workers were compared. The ability of the health worker to pick up the cause of death correctly can be termed as ‘sensitivity,’ and the ability to avoid assigning a wrong cause of death can be termed as ‘specificity’. Overall, when judged against the physician's diagnosis, the sensitivity was high for suicides (83%).

The main outcomes of this analysis were suicide rates by age and sex and the proportions of deaths in men and women attributed to suicide as the cause of death. Suicide rates, combined for sexes and separately for age group and gender were calculated by dividing the number of suicides by the number of individuals, as defined by the mid period population. The results were expressed as rates per 100,000 person years for the period between 1 July 2002 and 30 June 2007. The person years of observation were 798,695 of which 391,570 and 407,125 were for males and females, respectively. Proportions of suicides are expressed as the ratio of the number of suicide deaths to total number of deaths.

## RESULTS

The health workers reported 4720 deaths for the period 1 July 2002 to 30 June 2007. Of these deaths, 284 cases were classified as suicides. Table 1 provides the age-wise break up of the persons who committed suicide. Suicide was responsible for 6.6% of all deaths (7.3% in males and 5.8% in females). The median age of suicide was 42 years for males and 34 years for females and suicide rate was 44.7/100,000 for males and 26.8/100,000 for females. Male to female suicide ratio was 1.7. No family/mass suicides were reported in the period.

**Table 1:** Deaths due to suicide in various age groups in males and females

Age group	Male	Female	Combined
<14	2	5	7
15-24	20	33	53
25-34	37	17	54
35-44	31	16	47
45-54	25	5	30
55-64	32	10	42
65-74	22	12	34
>75	6	11	17
Total	175	109	284

**Figure 1:**
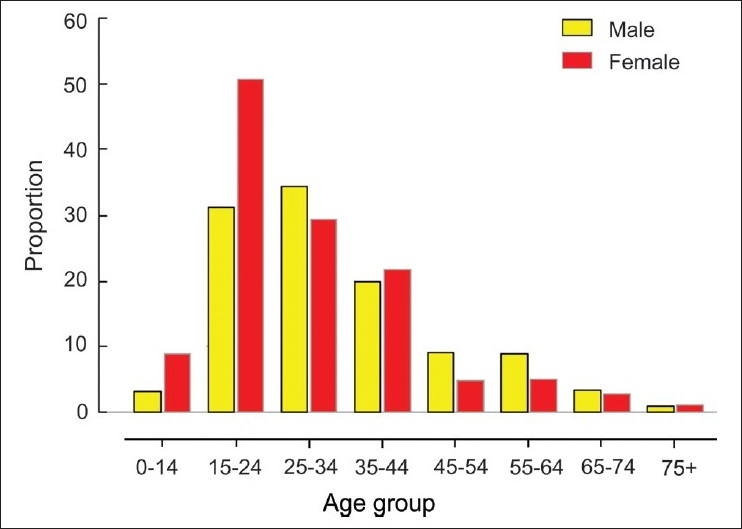
Contribution of suicide to all-cause deaths in relation to age and sex

Contribution of suicides to all cause-deaths in relation to age and sex is shown in Figure 1. Suicides constituted more than 50% of all deaths among females aged between 15 and 24 years. Out of 175 male suicides, 88 occurred in the age group of 15 to 44 years, while 66 deaths were suicides, out of 109 female deaths. Although suicide rates rose as age advanced, the relative contribution of suicide to all cause mortality fell as age advanced.

**Figure 2:**
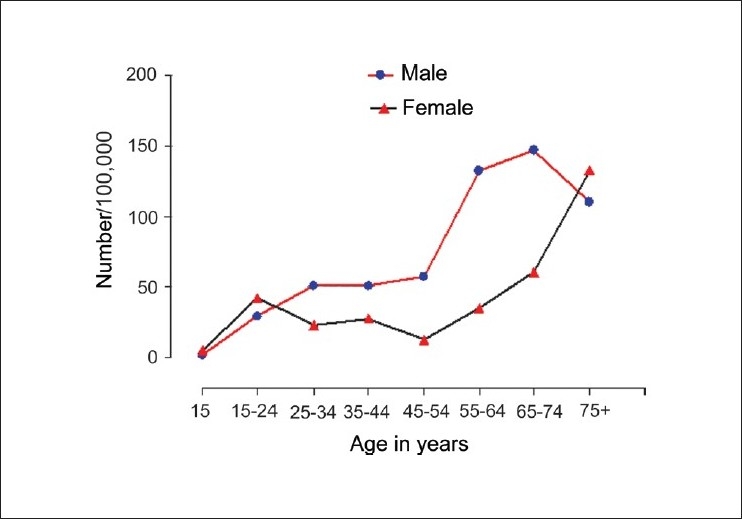
Age-specific rates of suicide among males and females

Age-specific death rates for suicide, averaged for the five-year period, are presented in Figure 2. Female suicide rates exceeded those of males in the very young and very old age groups. The rates diverged in favor of males from 24 years and declined after 74 years. Female suicide rates declined after 24 years reaching the lowest rate of 12.6/100,000 in women aged between 45 and 54 years. Women aged 75 or above had the highest suicide rates (132.6/100,000). Male to female suicide ratio varied from 0.4 in the youngest age group to 4.5 in the 45-54 year age groups.

Method of committing suicide was available for only 140 deaths. Hanging was by far the most frequently used method (64%), followed by poisoning (10%), drowning (9.3%), self-immolation/burning (6.4%), and jumping in front of trains (6.4%)

## DISCUSSION

Our analysis revealed that suicide rates in this rural community were higher than those reported by official agencies. However, the extent of under-reporting was much lower than those reported in Tamil Nadu. The rates that we report, in rural Kerala, are much lower than the Figures reported from rural Tamil Nadu. The observation that suicides contribute to the highest share of total deaths in young women between the ages 15 and 24 years is of great concern. It is clear that suicide rates vary in relation to age. However, the extent and pattern of change differs between males and females. In females, the suicide rate decreases after 24 years and reaches the lowest at 54 years. It rises again and peaks in women aged 75 or above. In males, the rate continues to rise steadily up to 74 years, showing a decline after 75 years.

**Figure 3:**
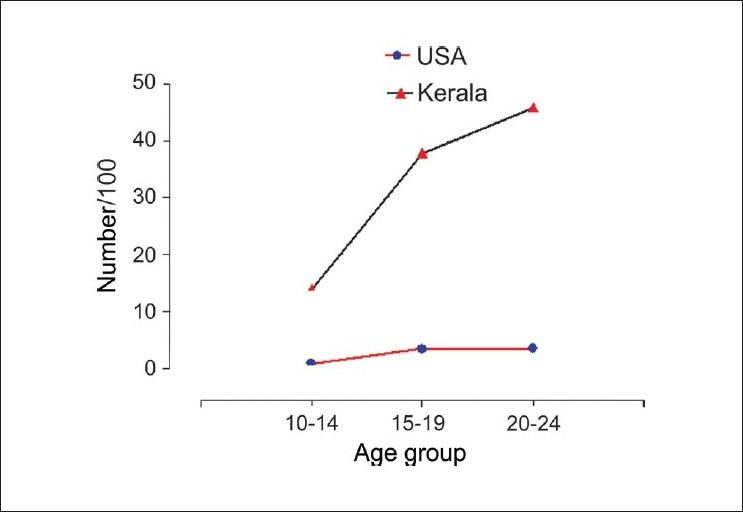
US data adapted from the National Vital Statistics System, United States, 2004[20]

Studies from rural Tamil Nadu have reported the highest suicide rates in the world. Suicide rates and risk of death from suicide vary widely and are dependent on individual, family, and societal level factors.[13–15] The increased rate of suicide among young women that we report, are consistent with the findings in the Tamil Nadu studies. The suicide rate for young women in the Kaniyambadi block was 152/100,000, while it was 109/100,000 in Villupuram.[5,7] Similarly, a higher rate of suicide among girls has also been reported from China and Singapore.[16,17] The variation in suicide rate across cultures and communities is also seen in younger age groups. Globally suicide rates among the youth are declining.[18] However, in the US, youth suicide rates have increased in recent times.[19] Suicide is the third leading cause of death among the youth in US, while in our community it is the leading cause of death. The suicide rate among young females that we report is 8 to 12 times higher than US rates for the same age groups [Figure 3].

The main strength of our study is the large population sample, with an age composition closely matching that of the Kerala population. Kerala has the highest rate of suicides reported among the major states in India and is best suited for any study on suicide. The use of resident health workers has ensured that even suicides that escaped reporting by official agencies were captured. Our study had certain limitations. This report is based on a comprehensive study of deaths, in which suicides formed only a part. The methods of suicide were collected only at the halfway stage. We acknowledge that the use of techniques like psychological autopsy could have furnished more information on the nature and cause of the suicides. With more training, the health workers will be able to gather more information on the causes and factors associated with suicide in this community.

Women of Kerala are the most educated in India and enjoy health standards comparable to that of developed countries.[21,22] We are surprised at the high suicide rates in women. A study on the factors associated with suicide in Tamilnadu reported recent adverse life events, interpersonal stress and relationship difficulties, severe financial distress, the use of alcohol, and issues related to gender, as risk factors.[23] Our analysis highlights the need to carry out further studies to understand the underlying causes of the high suicide rates in this apparently peaceful region.

Our results show that the level of under-reporting of suicides in rural Kerala is much less than that in Tamil Nadu. The absolute rates are also significantly lower than those reported in Tamil Nadu. In the context of our findings, we believe that the portrayal of the whole of South India as the suicide capital of the world is misleading. We recommend caution while extrapolating high rates of suicides reported from select communities to the state or national level.
